# The effect of TCF7L2 polymorphisms on inflammatory markers after 16 weeks of legume-based dietary approach to stop hypertension (DASH) diet versus a standard DASH diet: a randomised controlled trial

**DOI:** 10.1186/s12986-022-00671-7

**Published:** 2022-05-18

**Authors:** Somayeh Hosseinpour-Niazi, Parvin Mirmiran, Farzad Hadaegh, Maryam S. Daneshpour, Mehdi Hedayati, Fereidoun Azizi

**Affiliations:** 1 Nutrition and Endocrine Research Center, Research Institute for Endocrine Sciences, Shahid Beheshti University of Medical Sciences, No. 24, A’rabi St., Yeman Av., Velenjak, Tehran, 19395-4763 Iran; 2 Prevention of Metabolic Disorders Research Center, Research Institute for Endocrine Sciences, Shahid Beheshti University of Medical Sciences, Tehran, Iran; 3 Cellular and Molecular Research Center, Research Institute for Endocrine Sciences, Shahid Beheshti University of Medical Sciences, Tehran, Iran; 4 Endocrine Research Center, Research Institute for Endocrine Sciences, Shahid Beheshti University of Medical Sciences, Tehran, Iran

**Keywords:** Legumes, Type 2 diabetes, Inflammatory markers, Oxidative stress

## Abstract

**Backgrounds:**

This randomized controlled trial aimed to investigate the effects of replacing red meat with legumes in the dietary approach to stop hypertension (DASH) diet on inflammatory markers over 16 weeks in overweight and obese individuals with type 2 diabetes. Also, the modulatory effects of TCF7L2 rs7903146 variant on this effect were assessed.

**Methods:**

In this trial, 300 participants with type 2 diabetes, aged 30–65 years with an identified *TCF7L2* rs7903146 genotype, were studied. The participants were randomly assigned to the DASH diet or the legume-based DASH diet over 16 weeks. In the DASH diet group, the participants were instructed to follow the standard DASH diet. The legume-based DASH diet was similar to the standard DASH diet, with the exception that one serving of red meat was replaced with one serving of legumes at least five days a week. At the beginning of the study and 16-week follow-up, venous blood samples were collected from all participants who fasted for 12–14 h overnight. The serum concentration of High-sensitivity C-reactive protein (hs-CRP), tumor necrosis factor-α (TNF-α) and interleukin-6 (IL-6) was measured using an enzyme-linked immunosorbent assay (ELISA) kit. Also, the serum malondialdehyde (MDA) concentration was assessed based on a colorimetric method using a commercial kit. The primary outcome was the difference in hs-CRP changes between the diets. A secondary outcomes was the difference in IL-6, TNF-α, and MDA between the groups among total population and based on *TCF7L2* rs7903146 risk allele (CT + TT) and non-risk allele (CC) separately.

**Results:**

The hs-CRP level reduced in the legume-based DASH diet group as compared to the DASH diet group in the 16-week follow-up group. The levels of TNF-α, IL-6, and MDA reduced after the legume-based DASH diet relative to the DASH diet. Reduction of inflammatory markers was observed in both carriers of rs7903146 risk allele and non-risk allele.

**Conclusions:**

Substituting one serving of red meat with one serving of legumes in DASH diet, at least five days a week, could improve the hs-CRP, TNF-α, IL-6, and MDA in participants with type 2 diabetes regardless of having rs7903146 risk or non-risk allele.

*Trial registration* IRCT, IRCT20090203001640N17.

**Supplementary Information:**

The online version contains supplementary material available at 10.1186/s12986-022-00671-7.

## Introduction

Type 2 diabetes is a cause of long-term micro- and macrovascular complications [1]. These vascular complications are the main morbidities in individuals with type 2 diabetes [2]. Despite the focus on the management of conventional cardiovascular risk factors, such as dyslipidemia, hypertension, and dysglycemia to prevent morbidity and mortality in these patients, inflammation plays an important role in the development and progression of these disorders [3]. High-sensitivity C-reactive protein (hs-CRP), as a markers of inflammation, is independently associated with cardiovascular disease (CVD) and its risk factors [4, 5]. Reduction of hs-CRP leads to the risk reduction of micro- and macrovascular complications and decreases the mortality of CVD in patients with type 2 diabetes [3, 6].

The most common strategy for the management of inflammation in type 2 diabetes is a healthy diet [7]. One of the most challenging aspects of dietary modification for the prevention and management of cardiovascular risk factors, such as inflammation, is the selection of protein sources. Red meat is a major source of protein in most diets. However, the effect of red meat consumption on inflammation is ambiguous. Epidemiological studies have reported a positive association between red meat consumption and dietary patterns rich in red meat and inflammation and oxidative stress [8–12]. On the other hand, substituting red meat with alternative protein food sources (i.e., poultry, fish, legumes, or nuts) seems to have beneficial effects on the cardiometabolic risk factors [8]. However, there is no clear evidence on the beneficial effects of reducing red meat consumption and replacing red meat with other protein sources in clinical trials [13–18].

The effect of genetic factors on the risk of diabetes is estimated at 30–70% [19]. Transcription factor 7-like 2 (*TCF7L2*) gene rs7903146 polymorphism is a common type 2 diabetes-associated variant [20]. This polymorphism has been also associated with the progression of common complications of type 2 diabetes, especially CVD [21]. Impairment of hepatic insulin sensitivity and induction of insulin resistance are among molecular mechanisms through which *TCF7L2* rs7903146 variant increases the risk of type 2 diabetes and its most common comorbidity [22, 23]. Although the association between insulin resistance and abnormal secretion of inflammatory markers is well established [24, 25], the effect of *TCF7L2* gene on inflammatory markers and also the effect of interaction between environmental factors and this gene on inflammatory markers have been less investigated [26, 27]. Some studies documented that *TCF7L2* gene did not modify the effect of medications, such as fenofibrate, or diets rich in functional foods on inflammatory biomarkers [26, 28].

Considering the conflicting evidence on the effect of red meat consumption on inflammation, besides the limited number of studies on the modulatory effect of *TCF7L2* rs7903146 polymorphism on the relationship between diet and inflammation, the primary outcome of this randomized controlled trial was to investigate the effect of replacing red meat with legumes in the Dietary Approach to Stop Hypertension (DASH) diet on hs-CRP over 16 weeks in overweight and obese individual with type 2 diabetes. The secondary outcome of this study was to assess the effect of substituting red meat with legumes on other inflammatory markers, such as tumor necrosis factor-α (TNF-α) and interleukin-6 (IL-6), and oxidative stress markers including malondialdehyde (MDA). Another secondary outcome of this study was assessment of the effect of including legumes in the DASH diet on inflammatory biomarkers in *TCF7L2* rs7903146 risk allele (CT + TT) and non-risk allele (CC) carriers separately.

## Materials and methods

This randomized controlled trial was registered in the Iranian Registry of Clinical Trials (Trial registration: IRCT, IRCT20090203001640N17. Registered 20 may 2020, https://en.irct.ir/trial/46855). Ethical approval was obtained from the Ethics Committee of Research Institute for Endocrine Sciences of Shahid Beheshti University of Medical Sciences, Tehran, Iran (No. IR.SBMU.ENDOCRINE.REC.1399.001). All participants provided written informed consent forms before recruitment. According to the principles of the Declaration of Helsinki, the study procedures, purpose, and adverse events were explained to each participant (written and orally).

### Participants, randomization, and allocation concealment

This randomized controlled trial was conducted in the framework of Tehran Lipid and Glucose Study (TLGS). The TLGS is a large long-term community-based prospective study being conducted on a representative sample of residents from district No. 13 of Tehran, Iran. The population of this district represents the urban population of Tehran. The details of this study are described elsewhere [29]. Briefly, the first examination was initiated in March 1999. Using multistage stratified cluster random sampling, more than 15,000 individuals, aged ≥ 3 years, were enrolled. Overall, since 1999, the TLGS participants have undergone assessments for sociodemographic factors, lifestyle, medication use, socioeconomic status, anthropometric indices, and medical history of cardiovascular risk factors. The information is documented every three years in face-to-face visits by the local research team to update the previous data. Phases II, III, IV, V, and VI of this study were prospective follow-ups conducted during 2002–2004, 2005–2008, 2008–2011, 2012–2015, and 2016–2018, respectively.

Of 10,927 individuals participating in phase VI of the TLGS study, *TCF7L2* rs7903146 genotype was randomly determined in 8399 participants. Of these, 662 participants, aged 30–65 years, had type 2 diabetes with available information on *TCF7L2* rs7903146 genotype (CC genotype, n = 240; TT genotype, n = 128, and CT genotype, n = 294). The criteria for diagnosing diabetes included a fasting plasma glucose (FPG) level ≥ 126 mg/dL, two-hour plasma glucose ≥ 200 mg/dL, or using antihyperglycemic medications. Other inclusion criteria were being overweight or obese (BMI: 25–40 kg/m^2^); no weight changes in the last three months before enrollment; consumption of red meat ≥ 1 serving/d; and willingness to consume legumes in the diet. On the other hand, participants with pregnancy or lactation, cardiac, hepatic, or renal impairment (creatinine ≥ 1.4 mg/dL in men and ≥ 1.3 mg/dL in women), and insulin use were excluded.

**Fig. 1:**
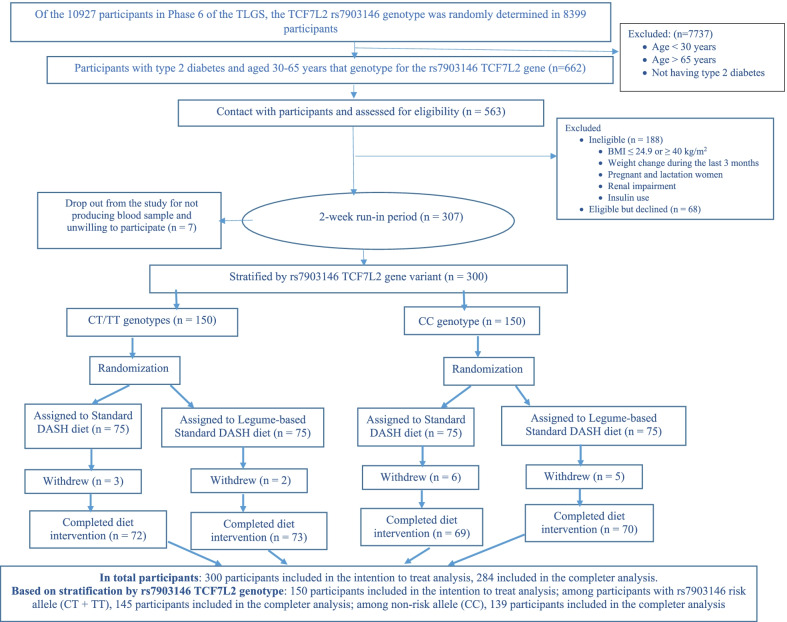
Flowchart for the participants

Randomization was carried out to generate the randomization sequence using the randomization website (www.randomization.com). The randomization sequence was separately generated for participants with *TCF7L2* rs7903146 risk allele (CT + TT) and non-risk allele (CC). Among participants eligible for this study, we selected 150 participants with genotype CC and 150 participants with genotype TT + CT and assigned them separately and randomly (1:1 ratio) to either the DASH diet group or the legume-based DASH diet group. The recruitment of participants is shown in Fig. 1.

The participants were randomly assigned to receive one of the two diets by a member of the TLGS staff. Using sealed and sequentially numbered opaque envelopes, the subject’s allocation to treatment was concealed from all staff members and the principal investigator; they were only opened sequentially by the dietitian in the presence of eligible participants in the first visit.

### Blinding

In the nutritional interventions, blinding of the participants was practically impossible. However, the participants were unaware of their assignment to the intervention groups before enrolment. Individuals who assessed the outcomes, including laboratory technicians and staff members, were blind to the participants’ assignment, and dietary interventions were provided by a dietitian.

### Dietary interventions

First, 307 individuals participated in a two-week run-in period when they consumed their usual diet while eliminating the legume intake. In the run-in period, seven participants were unwilling to continue the study and were excluded. At the end of the run-in period, 300 participants, stratified based on risk and non-risk alleles, were randomly assigned to the DASH diet group or legume-based DASH diet group over 16 weeks.

The participants’ energy requirements were estimated from the resting energy expenditure, based on the Mifflin-St Jeor formula and multiplied by the physical activity coefficient [30]. Because the participants were overweight and obese, 500–700 kcal/d was deducted from their energy requirements. Based on each participant's energy requirement, the dietitian determined the diet, which contained approximately 25–30% fat, 15% protein, and 55–60% carbohydrate. In the DASH diet group, the participants were instructed to follow the DASH diet (2000–3000 kcal based on the participant’s energy requirement), composed of 8–12 servings/day of fruits and vegetables, 7–15 servings of whole grains, 2–3 servings of low-fat dairy products, two servings of red meat, one serving of nuts and seeds, and limited intake of sweets (five servings per week). The legume-based DASH diet was similar to the standard DASH diet, with the exception that one serving of red meat was replaced with one serving of legumes at least five days a week. Also, because legumes are equivalent to one serving of whole grains, one serving of bread was also deducted from the legume-based DASH diet. In both diets, the participants were advised to consume less than or equal to one teaspoon of salt per day (2300 mg/d).

To evaluate the participants’ adherence to the interventions, the dietitian instructed them to record their daily dietary intake using a three-day food record (two weekdays and one weekend day) every weeks. The dietitian called the participants every week to gather their dietary information, compared their information with the instructed diet, and reinforced their diet adherence. The intake of macro- and micronutrients was also calculated using NUTRITIONIST III Version 7.0 (N-Squared Computing, Salem, OR, USA), designed for Iranian foods. All participants were requested to maintain their physical activity and not to change their medications during the 16-week interventions unless prescribed by their physicians.

### Primary and secondary outcomes

The primary outcome of this study was the difference in hs-CRP change from baseline to week 16 of follow-up between the groups. The secondary outcome was the difference in IL-6, TNF-α, and MDA changes between the groups. Another secondary outcome was assessment of changes in the inflammatory markers, based on *TCF7L2* rs7903146 risk allele (CT + TT) and non-risk allele (CC) separately.

### Measurements

Weight was measured using a digital scale (Seca 707; range: 0–150 kg; Seca GmbH, Germany), with minimal clothing and without shoes; it was recorded to the nearest 100 g. Height was also measured in a standing position, with shoulders in neutral alignment using a stadiometer (Seca 225; Seca GmbH, Germany) without shoes and recorded to the nearest 0.5 cm. The body mass index (BMI) was calculated by dividing weight in kilograms by height in meters squared.

At the beginning of the study and 16-week follow-up (between 7∶00 a.m. and 9∶00 a.m.), venous blood samples were collected from all participants who fasted for 12–14 h overnight. The blood samples were placed in vacutainer tubes, centrifuged within 30–45 min of collection, and stored in a − 80 °C freezer. The serum concentration of hs-CRP was measured using an enzyme-linked immunosorbent assay (ELISA) kit (ZellBio, Germany). To analyze the concentrations of TNF-α and IL-6, ELISA assay was performed using a commercial kit (Diaclone, France). Also, the serum MDA concentration was assessed based on a colorimetric method using a commercial kit (ZellBio GmbH, Ulm, Germany). The intra-assay coefficients of variation (CVs) for the serum hs-CRP, IL-6, TNF-α, and MDA were 2.7%, 8.1%, 6.3%, and 3.3%, respectively.

Genomic DNA was extracted from the buffy coat of samples, using a standard salting out method with proteinase K. The quality of extracted DNA was determined using the NanoDrop 1000 Spectrophotometer. Samples in the range of 1.7 < A260/A280 < 2 were excluded due to low quality and concentration. DNA samples were processed on a HumanOmniExpress-24-v1-0 BeadChip (containing 649,932 SNP loci with an average mean distance of 4 kb) at deCODE Genetics Company (Reykjavik, Iceland), according to the manufacturer’s instructions (Illumina Inc., San Diego, CA, USA). Quality control procedures were also performed using PLINK V. 1.07 and R Statistic V. 3.2 [31].

### Assessment of other variables

Medication regimen (e.g., antihypertensive, lipid-lowering, and anti-diabetes drugs and others) and supplement intake were collected. Physical activity was also assessed using the Modifiable Activity Questionnaire (MAQ), and the frequency and amount of time spent per week on physical activity over the last year were recorded.

### Statistical analysis

The target sample size was measured to be 150 in each intervention group to detect a difference of 1 mg/dl reduction in hs-CRP [32] between the two diets in the total population by assuming an α error of 0.05, a β error of 0.20, power of 80%, and an attrition rate of 20%.

Through visual inspection of the histograms, scatter plots, and Shapiro–Wilk test, the normal distribution of data was assessed. Normal variables are presented as mean ± SD for demographic variables and mean ± SEM for dietary variables. Skewed variables are also presented as median (interquartile range) and dichotomous variables as count (percentage). Analyses were performed according to the per-protocol and intention-to-treat principles. Multiple imputation by chained equations method was also applied to impute the primary and secondary missing outcomes. In this method, the predictors included all variables presented in Table 1, *TCF7L2* rs7903146 variant, and intervention diets.

**Table 1:** Baseline characteristic of participants according to group of intervention diets and TCF7L2 rs7903146 gene variant

	Total population		CC genotypes		TT + CT genotypes	
	DASH diet	Legume-based DASH diet	DASH diet	Legume-based DASH diet	DASH diet	Legume-based DASH diet
Participants, n	150	150	75	75	75	75
Age, years	55.5 (6.9)	55.4 (7.1)	55.1 (6.7)	55.2 (8.1)	55.6 (7.1)	55.5 (6.1)
Female, n (%)	85 (56.3)	86 (57.7)	42 (56.0)	45 (60.0)	43 (56.6)	41 (55.4)
hsCRP, mg/dl	3.6 (1.9–5.0)	3.7 (2.1–5.0)	3.6 (2.1–5.4)	3.7 (1.9–4.8)	3.5 (1.9–4.6)	3.8 (2.1–5.2)
MDA, µM	5.1 (3.7–7.7)	5.0 (3.6–8.3)	5.0 (3.7–7.6)	5.1 (3.4–8.3)	5.1 (3.4–8.3)	4.8 (3.6–7.9)
TNF-α, pg/ml	13.0 (11.2–15.9)	11.4 (9.8–15.8)	11.4 (9.8–13.4)	11.4 (9.8–13.4)	15.2 (12.6–17.9)	11.4 (9.9–16.5)
IL-6, pg/ml	4.3 (3.7–5.4)	3.8 (3.5–4.6)	3.9 (3.6–5.2)	3.7 (3.4–4.7)	4.5 (4.1–5.7)	3.9 (3.5–4.6)
Obese, n (%)	72 (48.0)	74 (49.3)	42 (56.0)	41 (54.7)	30 (40.0)	33 (44.0)
Physical activity levels, Met h/week	3.5 (2.7)	3.3 (2.7)	3.6 (2.8)	3.4 (2.3)	3.3 (2.6)	3.4 (3.0)
Academic degree, n (%)	18 (11.9)	22 (14.8)	6 (8.0)	6 (8.0)	12 (15.8)	16 (21.6)
Medication						
Antihyperglycemic Medications						
Metformin, n (%)	55 (36.7)	69 (46.0)	32 (42.7)	39 (52.0)	23 (30.7)	30 (40.0)
Sulfonylurea, n (%)	40 (26.5)	29 (19.3)	21 (28.0)	14 (18.7)	19 (25.3)	15 (20.0)
Metformin + sulfonylurea, n (%)	45 (30.0)	31 (20.7)	20 (26.7)	13 (17.3)	25 (33.3)	18 (24.0)
Metformin + thiazolidinedione, n (%)	5 (3.3)	11 (7.3)	2 (2.7)	9 (12.0)	3 (4.0)	2 (2.7)
Others, n (%)	5 (3.3)	10 (6.7)	0 (0)	0 (0)	5 (6.7)	10 (13.3)
Lipid lowering drugs						
Statin use, n (%)	86 (57.0)	84 (56.4)	47 (62.7)	42 (56.0)	39 (51.3)	43 (57.3)
Others, n (%)	1 (0.7)	4 (2.7)	0 (0.0)	2 (2.7)	1 (1.3)	2 (2.7)
Antihypertensive drugs						
ACE inhibitor/ARB use, n (%)	52 (34.7)	48 (32.0)	28 (37.3)	26 (34.7)	24 (32.0)	22 (29.3)
Thiazide, n (%)	7 (4.7)	2 (1.3)	3 (4.0)	1 (1.3)	4 (5.3)	1 (1.3)
Others, n (%)	10 (6.7)	8 (5.3)	8 (10.7)	4 (5.3)	2 (2.7)	4 (5.3)
Asprin n (%)	28 (18.7)	28 (18.7)	11 (14.6)	13 (17.3)	17 (22.7)	15 (20.0)
Supplement						
Vitamin E	1 (0.7)	0 (0%)	0 (0%)	0 (0%)	1 (1.3%)	0 (0%)
Vitamin D	32 (21.3)	34 (22.7)	17 (22.7)	18 (24.0)	15 (20.0)	16 (21.3)
Vitamin B complex	17 (11.3)	15 (10.0)	7 (9.3)	9 (12.0)	10 (13.3)	6 (8.0)
W-3 PUFA fatty acids	4 (2.7)	3 (2.0)	3 (4.0)	0 (0.0)	1 (1.3)	3 (4.0)

Obese BMI ≥ 30 kg/m2

Data are mean (SD) or median (interquartile range) unless otherwise indicated

Differences in the changes of outcomes between the two diets in the total population and also with respect to the *TCF7L2* rs7903146 risk allele (CT + TT) and non-risk allele (CC) were compared using the analysis of covariance (ANCOVA). Model 1 was adjusted for the baseline values, and model 2 was further adjusted for oral antihyperglycemic Medications. The gene-diet interaction was analyzed by an ANCOVA multivariate interaction model, and P-value < 0.2 indicated the significant effect of gene-diet interaction on the outcomes. The Benjamini–Hochberg correction method for multiple testing yielded critical P-values of < 0.1 for the secondary outcome comparisons. Cohen’s d for effect size (0.20, 0.50, and 0.80 interpreted as small, medium, and large treatment effects, respectively) was also calculated based on mean and SD [33]. All analyses were performed in Stata Version 14.0 (StataCorp LLC, TX, USA).

## Results

This trial was carried out between July 11, 2020 and March 10, 2021. Of 563 participants screened for eligibility, 300 were randomly allocated to the diet groups. Sixteen participants withdrew from the study, and finally, 284 individuals completed the study (Fig. 1). The mean age and BMI of the participants were 55.4 years (SD = 7.0) and 30.4 kg/m^2^ (SD = 3.4), respectively (42.9% female and 48.7% obese). No significant differences were found in the baseline variables, except for the use of oral antihyperglycemic Medications, between the two groups in the total population and also among rs7903146 risk allele (CT + TT) and non-risk allele (CC) carriers (Table 1). Compared to the DASH diet, metformin was more commonly used for the legume-based DASH diet group (46.0 vs. 36.7), while participants in the DASH diet group were more treated with metformin plus sulfonylurea (20.7 vs. 30.0). Among carriers of rs7903146 non-risk allele, metformin and metformin plus thiazolidinedione was used slightly more often for participants in the legume-based DASH diet group, while the DASH diet group was treated more with sulfonylurea, and metformin plus sulfonylurea. Metformin was slightly more often used for the carriers of rs7903146 T allele in the legume-based DASH diet group, while the DASH diet group was treated more with metformin plus sulfonylurea.

Analysis of the subjects’ food records showed that compared to the DASH diet, the intake of legumes and fiber was higher, while the intake of red meat and cholesterol was lower in the legume-based DASH diet group. No significant difference was found regarding the total energy requirements, macronutrients, and dietary food groups between the groups (Additional file 1: Table 1).

### Primary outcome

The ITT and completer analyses yielded similar findings (Additional file 1: Table 2); therefore, only the ITT findings are reported. The hs-CRP level was reduced at week 16 in the legume-based DASH diet group compared to the DASH diet group (mean difference of change: -0.58 mg/dL [− 0.78 to − 0.39] in the DASH diet group vs. − 1.20 mg/dL [− 1.39 to −1.01] in the legume-based DASH diet group; Cohen’s d = 0.41 [0.64–0.18]) after adjustment for the baseline values and oral antihyperglycemic Medications (model 2). This reduction was observed in both carriers of rs7903146 risk allele and non-risk allele (Table 2).

**Table 2:** The 16-week change in inflammatory and oxidative stress markers and anthropometric measures after the DASH diet and legume-based DASH diet according to TCF7L2 rs7903146 gene variant

	Total population				CC genotype				CT + TT genotype				
	DASH diet	Legume-based DASH diet	*P value*	q value	DASH diet	Legume-based DASH diet	*P value*	*q value*	DASH diet	Legume-based DASH diet	*P value*	*q value*	*P i*
*Primary outcome*													
**hsCRP (mg/dl)**													
Model 1	− 0.57 (− 0.76 to − 0.38)	− 1.22 (− 1.40 to − 1.02)	< 0.001		− 0.51 (− 0.87 to − 0.15)	− 1.08 (− 1.27 to − 0.88)	0.001		− 0.65 (− 0.95 to − 0.34)	− 1.33 (− 1.53 to − 1.14)	0.002		0.421
Model 2	− 0.58 (− 0.78 to − 0.39)	− 1.20 (− 1.39 to − 1.01)	< 0001		− 0.51 (− 0.87 to − 0.15)	− 1.08 (− 1.28 to − 0.88)	0.002		− 0.65 (− 0.95 to − 0.35)	− 1.34 (− 1.52 to − 1.15)	0.002		0.237
*Secondary outcomes*													
**MDA (µM)**													
Model 1	− 1.03 (− 1.28 to − 0.78)	− 1.64 (− 1.88 to − 1.38)	< 0.001	0.016	− 0.91 (− 1.24 to − 0.58)	− 1.58 (− 1.97 to − 1.18)	0.016	0.040	− 1.11 (− 1.43 to − 0.81)	− 1.73 (− 2.11 to − 1.35)	0.021	0.040	0.455
Model 2	− 1.02 (− 1.27 to − 0.77)	− 1.64 (− 1.89 to − 1.39)	< 0.001	0.016	− 0.91 (− 1.24 to − 0.58)	− 1.58 (− 1.97 to − 1.18)	0.016	0.040	− 1.11 (− 1.43 to − 0.80)	− 1.73 (− 2.11 to − 1.35)	0.023	0.040	0.573
**TNF**− **α (pg/ml)**													
Model 1	− 0.76 (− 1.23 to − 0.29)	− 2.04 (− 2.51 to − 1.57)	< 0.001	0.016	− 0.68 (− 1.28 to − 0.08)	− 1.78 (− 2.41 to − 1.16)	0.010	0.038	− 0.87 (− 1.63 to − 0.11)	− 2.27 (− 2.97 to − 1.58)	0.006	0.032	0.593
Model 2	− 0.77 (− 1.24 to − 0.29)	− 2.04 (− 2.51 to − 1.56)	< 0.001	0.016	− 0.68 (− 1.45 to − 0.08)	− 1.78 (− 2.42 to − 1.14)	0.010	0.038	− 0.87 (− 1.60 to − 0.14)	− 2.28 (− 2.98 to − 1.57)	0.012	0.038	0.496
**IL**− **6 (pg/ml)**													
Model 1	− 0.53 (− 0.70 to − 0.36)	− 0.93 (− 1.10 to − 0.76)	0.001	0.016	− 0.52 (− 0.68 to − 0.36)	− 0.82 (− 1.05 to − 0.58)	0.028	0.044	− 0.52 (− 0.82 to − 0.22)	− 1.06 (− 1.28 to − 0.85)	0.019	0.041	0.753
Model 2	− 0.51 (− 0.68 to − 0.34)	− 0.95 (− 1.12 to − 0.78)	0.004	0.032	− 0.52 (− 0.68 to − 0.36)	− 0.81 (− 1.04 to − 0.58)	0.012	0.038	− 0.52 (− 0.82 to − 0.22)	− 1.06 (− 1.28 to − 0.85)	0.019	0.040	0.793
**BMI (kg/m**^**2**^**)**													
Model 1	− 1.48 (− 1.60 to − 1.36)	− 1.57 (− 1.69 to − 1.45)	0.287	0.382	− 1.41 (− 1.59 to − 1.23)	− 1.56 (− 1.73 to − 1.39)	0.238	0.346	− 1.54 (− 1.67 to − 1.42)	− 1.58 (− 1.79 to − 1.37)	0.709	0.756	0.617
Model 2	− 1.49 (− 1.61 to − 1.37)	− 1.56 (− 1.68 to − 1.44)	0.424	0.492	− 1.41 (− 1.59 to − 1.23)	− 1.56 (− 1.73 to − 1.39)	0.431	0.492	− 1.54 (− 1.67 to − 1.42)	− 1.58 (− 1.79 to − 1.37)	0.859	0.859	0.859

DASH, dietary approach to stop hypertension; FPG, fasting plasma glucose; WC, waist circumference; HOMA-IR, homeostatic model assessment for insulin resistance; Pi, P for interaction between TCF7L2 rs7903146 gene variant and intervention diets

Data for change in primary and secondary outcomes are express as mean (95% confidence interval)

Model 1 adjusted for baseline values

Model 2 adjusted for baseline values and oral anti-diabetic medications

P values were calculated by ANCOVA

q value were calculated by Benjamini–Hochberg correction and Q < 0.2 is significant

### Secondary outcomes

After adjustments for the baseline variables and oral antihyperglycemic Medications, TNF-α (− 0.77 pg/mL [− 1.24 to − 0.29] in the DASH diet vs. -2.04 pg/mL [− 2.51 to − 1.56] in the legume-based DASH diet), IL-6 (− 0.51 pg/mL [− 0.68 to − 0.34] in the DASH diet vs. − 0.95 pg/mL [− 1.12 to − 0.78] in the legume-based DASH diet), and MDA (− 1.02 µM [− 1.27 to − 0.77] in the DASH diet vs. − 1.64 µM [− 1.89 to − 1.39] in the legume-based DASH diet) reduced after the legume-based DASH diet intervention compared to the DASH diet; this reduction was observed in both risk allele and non-risk allele carriers. However, BMI did not change significantly after adherence to the legume-based DASH diet as compared to the DASH diet (Table 2).

## Discussion

In this weight-loss interventional trial among individual with type 2 diabetes, the inflammatory and oxidative stress status improved by replacing one serving of red meat with legumes in the DASH diet at least five days a week, regardless of having rs7903146 risk or non-risk allele.

Generally, low-grade inflammation occurs in the insulin resistance stage of type 2 diabetes. Therefore, identifying dietary determinants that increase inflammation and replacing them with dietary food groups that reduce inflammation are important. In many observational studies, but not all [9, 13], a positive association has been observed between biomarkers of inflammation and red meat consumption or red meat-rich dietary patterns [8, 10–12]. Also, substitution of a serving of total red meat with high-quality plant protein sources, such as legumes, was associated with lower CRP concentrations [8]. However, there is no clear evidence on the effects of changes in red meat intake and replacement of red meat with other protein sources on inflammation biomarkers in dietary interventions. Although substituting carbohydrates with proteins in interventional studies has shown no effects on inflammatory markers [16, 17, 34, 35], the results related to the substitution of red meat with other protein sources are controversial. High-protein diets, both animal and plant proteins, have been suggested to reduce inflammatory markers [18]. In a previous study, although replacement of pork with chicken and red meat did not change the CRP concentration [36], partial replacement of red meat with plant proteins, such as soy protein and legumes, improved the inflammatory biomarkers [37, 38]. Moreover, replacement of red meat with 30 g of soy was adequate in reducing the concentration of inflammatory markers in postmenopausal women [38, 39]. In the current study, improvement of inflammatory markers was achieved by replacement of one serving of red meat with legumes at least five days a week; this finding is consistent with previous clinical trials and a systematic review and meta-analysis, documenting the health-promoting effects of legume intake, with a median intake of 63 g/d to 150 g (~ 1½ servings/d) [37, 40–42]. This effect might be due to the higher consumption of dietary fiber and low-glycemic-load carbohydrates in diets with high-quality plant protein sources.

The *TCF7L2* rs7903146 SNP is the most important genetic predictor of T2DM [20]. Disturbances in insulin sensitivity and induction of insulin resistance are among the molecular mechanisms through which *TCF7L2* rs7903146 variant increases the risk of T2DM [43]. Although the role of insulin resistance in inducing inflammation has been identified [25], the effect of *TCF7L2* gene on inflammation has been less investigated [26, 27, 44]. There was no significant difference in the inflammatory markers among *TCF7L2* genotypes after treatment with fenofibrate [26]. Also, dietary patterns rich in functional foods did not modify the effects on CRP [28].

Although few studies have examined the modulatory effect of *TCF7L2* rs7903146 variant on the relationship between diet and inflammation, some studies have reported that the detrimental effects of *TCF7L2* gene on cardiometabolic risk factors may be improved by anti-inflammatory dietary patterns, such as the Mediterranean diet [45, 46]. The DASH dietary pattern is another anti-inflammatory diet. Although the beneficial effect of DASH diet on the management of cardiovascular risk factors, such as inflammation, has been documented [47–49], to the best of our knowledge, no study has yet investigated the effect of interaction between the DASH diet and *TCF7L2* gene on these risk factors.

Previous studies, however, have assessed the interaction between the DASH diet and some genes, such as genetic predisposition to obesity, based on BMI-associated variants [50, 51], as well as MC4R rs17782313 polymorphism [52] on cardiometabolic risk factors. In the Nurses’ Health Study and the Health Professionals Follow-up Study, during a 20-year follow-up, the effect of interaction between adherence to DASH diet and genetic predisposition to obesity (based on 77 SNPs) on changes in body weight was reported [50]. The detrimental effect of genetic predisposition on weight gain was reduced by greater adherence to the DASH diet; this effect was more pronounced among participants with a higher genetic risk of obesity [50]. In another study investigating three observational cohorts of US women and men, adherence to the DASH diet accentuated the detrimental effects of the genetic risk score (GRS), based on 97 BMI-associated variants, on BMI [51]. Also, in a cross-sectional study, high adherence to the DASH diet modified the effect of melanocortin-4 receptor (MC4R) rs17782313 polymorphism on cardiometabolic risk factors, including triglyceride concentration, blood pressure, and glucose concentration, especially among MC4R rs17782313 risk allele carriers [52]. Nevertheless, in the current study, we did not find any effect of interaction between the diet and rs7903146 variant on inflammatory markers in individual with type 2 diabetes. Our findings must be examined in other ethnic populations with different allele frequencies; also, family-based investigations on a large sample size are needed.

Some limitations of the current study need to be addressed. The assessment of adherence to dietary interventions was based on self-report diet records, and because of our limited funding, we could not measure the biochemical index of adherence to dietary interventions. Therefore, the dietitian called the participants once a week and encouraged their adherence to the dietary recommendations. Another limitation of this study was not blinding the participants to the study objectives, which might have affected the subjects’ behaviors. Finally, this study was conducted in an area with a middle to high socioeconomic status, and our findings cannot be extrapolated to individual with type 2 diabetes with a low socioeconomic status.

In conclusion, substituting one serving of red meat with one serving of legumes in DASH diet, at least five days a week, could improve the hs-CRP, TNF-α, IL-6, and MDA in participants with type 2 diabetes regardless of having rs7903146 risk or non-risk allele.

## Supplementary Information

**Additional file 1.** Dietary intake of the participants according to intervention diets and TCF7L2 rs7903146 gene variant.

## Data Availability

The datasets generated and/or analysed during the current study are not publicly available due institution’s policy but are available from the corresponding author on reasonable request.

## Abbreviations

hs-CRP: C-reactive protein
CVD: Cardiovascular disease
*TCF7L2*: Transcription factor 7-like 2
DASH: Dietary approach to stop hypertension
TNF-α: Tumor necrosis factor-α
IL-6: Interleukin-6
MDA: Malondialdehyde
TLGS: Tehran Lipid and Glucose Study
ELISA: Enzyme-linked immunosorbent assay
ANCOVA: Analysis of covariance
MC4R: Melanocortin-4 receptor
