# Supplementary material for: The effect of TCF7L2 polymorphisms on inflammatory markers after 16 weeks of legume-based dietary approach to stop hypertension (DASH) diet versus a standard DASH diet: a randomised controlled trial
Source: Nutr Metab (Lond). 2022 May 18;19:35. doi: 10.1186/s12986-022-00671-7 (PMC9118794; doi:10.1186/s12986-022-00671-7)
Supplement: Supplementary file 1 — Additional file 1. Dietary intake of the participants according to intervention diets and TCF7L2 rs7903146 gene variant. [file 12986_2022_671_MOESM1_ESM.docx]

| Supplementary Table 1. Dietary intake of the participants according to intervention diets and TCF7L2 rs7903146 gene variant | | | | | | | | |
| --- | --- | --- | --- | --- | --- | --- | --- | --- |
|  | Total population | |  | CC genotypes | |  | TT+ CT genotypes | |
| ***Dietary food groups*** | DASH diet | Legume-based DASH diet |  | DASH diet | Legume-based DASH diet |  | DASH diet | Legume-based DASH diet |
| Legumes *(serving/day)* | 0.2 (0.02) | 1.2 (0.02) |  | 0.2 (0.03) | 1.2 (0.03) |  | 0.2 (0.03) | 1.2 (0.03) |
| Red meat *(serving/day)* | 2.2 (0.02) | 1.2 (0.02) |  | 2.2 (0.03) | 1.2 (0.03) |  | 2.2 (0.03) | 1.3 (0.03) |
| Poultry and fish *(serving/day)* | 0.8 (0.03) | 0.3 (0.03) |  | 0.8 (0.04) | 0.3 (0.04) |  | 0.8 (0.04) | 0.3 (0.04) |
| Whole grain *(serving/day)* | 11.9 (0.5) | 11.8 (0.5) |  | 12.2 (0.8) | 11.4 (0.8) |  | 11.6 (0.8) | 12.2 (0.8) |
| Refined grain *(serving/day)* | 2.8 (0.2) | 2.5 (0.2) |  | 3.2 (0.3) | 2.4 (0.3) |  | 2.4 (0.3) | 2.6 (0.3) |
| Fruit *(serving/day)* | 5.2 (0.3) | 5.6 (0.3) |  | 6.6 (0.4) | 5.8 (0.4) |  | 5.6 (0.4) | 5.4 (0.4) |
| Vegetable *(serving/day)* | 6.3 (0.2) | 6.5 (0.2) |  | 4.4 (0.3) | 6.3 (0.3) |  | 6.2 (0.3) | 6.8 (0.3) |
| Nuts and seed *(serving/day)* | 1.2 (0.03) | 1.3 (0.03) |  | 1.2 (0.04) | 1.3 (0.04) |  | 1.2 (0.04) | 1.2 (0.04) |
| Low fat dairy products *(serving/day)* | 2.3 (0.08) | 2.4 (0.08) |  | 2.3 (0.1) | 2.4 (0.1) |  | 2.2 (0.1) | 2.3 (0.1) |
| High fat dairy products *(serving/day)* | 0.5 (0.08) | 0.3 (0.08) |  | 0.5 (0.1) | 0.2 (0.1) |  | 0.4 (0.1) | 0.3 (0.1) |
| Fats and oils*(serving/day)* | 5.8 (0.05) | 5.7 (0.05) |  | 5.6 (0.07) | 5.6 (0.07) |  | 5.9 (0.07) | 5.8 (0.07) |
| Sweets *(serving/day)* | 1.1 (0.1) | 1.0 (0.1) |  | 1.2 (0.2) | 0.9 (0.2) |  | 0.8 (0.2) | 1.0 (0.2) |
| *Macronutrient intake )* |  |  |  |  |  |  |  |  |
| Energy (kcal) | 2324 (58) | 2225 (58) |  | 2326 (82) | 2188 (82) |  | 2322 (82) | 2262 (82) |
| Carbohydrate (% energy) | 56.9 (0.6) | 56.5 (0.6) |  | 57.1 (0.9) | 56.7 (0.9) |  | 57.0 (0.9) | 56.0 (0.9) |
| Total fat (% energy) | 30.1 (0.6) | 29.6 (0.6) |  | 29.8 (0.8) | 29.3 (0.8) |  | 30.4 (0.8) | 29.8 (0.8) |
| Protein (% energy) | 13.3 (0.2) | 13.3 (0.2) |  | 13.4 (0.3) | 13.1 (0.3) |  | 13.3 (0.3) | 13.2 (0.3) |
| Fiber (g/d) | 33.4 (1.6) | 41.3 (1.6) |  | 31.8 (2.3) | 41.5 (2.3) |  | 35.1 (2.3) | 41.1 (2.3) |
| Cholesterol (g/d) | 208 (9) | 184 (8) |  | 214 (12) | 182 (12) |  | 202 (12) | 186 (12) |

Dietary intake include the mean intake of twenty-four food records during 16 weeks follow-up.

Data are mean (SEM)

| Supplementary Table 2. The 16-week change in inflammatory and oxidative stress markers and anthropometric measures after the DASH diet and legume-based DASH diet according to TCF7L2 rs7903146 gene variant based on intention to treat analysis | | | | | | | | | | | | | |
| --- | --- | --- | --- | --- | --- | --- | --- | --- | --- | --- | --- | --- | --- |
|  | Total population | | |  | CC genotype | | | | CT+TT genotype | | | |  |
|  | DASH diet | Legume-based DASH diet | *P value* | q  value | DASH diet | Legume-based DASH diet | *P value* | *q value* | DASH diet | Legume-based DASH diet | *P value* | *q value* | *P i* |
| *Primary outcome* | | | | | | | | | | | | |  |
| **hsCRP (ng/dl)** |  |  |  |  |  |  |  |  |  |  |  |  |  |
| Model 1 | -0.59  (-0.77 to -0.42) | -1.24  (-1.42 to -1.07) | <0.001 |  | -0.61  (-0.90 to -0.32) | -1.06  (-1.25 to -0.86) | <0.001 |  | -0.65  (-0.96 to -0.33) | -1.36  (-1.56 to -1.16) | 0.002 |  | 0.494 |
| Model 2 | -0.60  (-0.78 to -0.43) | -1.23  (-1.41 to -1.06) | <0001 |  | -0.61  (-0.91 to -0.32) | -1.06  (-1.26 to -0.86) | <0.001 |  | -0.65  (-0.95 to -0.34) | -1.36  (-1.55 to -1.17) | 0.003 |  | 0.292 |
| *Secondary outcomes* | | | | | | | | | | | | |  |
| **MDA (µM)** |  |  |  |  |  |  |  |  |  |  |  |  |  |
| Model 1 | -1.03  (-1.28 to -0.78) | -1.63  (-1.88 to -1.38) | <0.001 | 0.015 | -0.91  (-1.24 to -0.58) | -1.58  (-1.97 to -1.18) | 0.016 | 0.034 | -1.11  (-1.43 to -0.81) | -1.73  (-2.11 to -1.35) | 0.021 | 0.038 | 0.732 |
| Model 2 | -1.03  (-1.28 to -0.78) | -1.64  (-1.89 to -1.39) | <0.001 | 0.015 | -0.91  (-1.24 to -0.58) | -1.58  (-1.97 to -1.18) | 0.016 | 0.034 | -1.11  (-1.43 to -0.80) | -1.73  (-2.11 to -1.35) | 0.023 | 0.038 | 0.862 |
| **TNF-α (pg/ml)** |  |  |  |  |  |  |  |  |  |  |  |  |  |
| Model 1 | -0.88  (-1.29 to -0.47) | -2.21  (-2.62 to -1.80) | <0.001 | 0.015 | -0.86  (-1.44 to -0.29) | -1.96  (-2.43 to -1.49) | 0.002 | 0.015 | -0.96  (-1.74 to -0.17) | -2.38  (-2.81 to -1.95) | 0.002 | 0.015 | 0.694 |
| Model 2 | -0.91  (-1.32 to -0.50) | -2.18  (-2.59 to -1.77) | <0.001 | 0.015 | -0.86  (-1.45 to -0.28) | -1.96  (-2.44 to -1.48) | 0.004 | 0.015 | -0.96  (-1.71 to -0.21) | -2.38  (-2.81 to -1.96) | 0.003 | 0.015 | 0.475 |
| **IL-6 (pg/ml)** |  |  |  |  |  |  |  |  |  |  |  |  |  |
| Model 1 | -0.56  (-0.71 to -0.42) | -0.98  (-1.12 to -0.83) | <0.001 | 0.015 | -0.54  (-0.69 to -0.39) | -0.89  (-1.08 to -0.70) | 0.004 | 0.015 | -0.56  (-0.83 to -0.30) | -1.08  (-1.26 to -0.90) | 0.009 | 0.022 | 0.863 |
| Model 2 | -0.55  (-0.69 to -0.40) | -0.99  (-1.14 to -0.85) | <0.001 | 0.015 | -0.54  (-0.70 to -0.39) | -0.89  (-1.08 to -0.70) | 0.002 | 0.015 | -0.56  (-0.83 to -0.31) | -1.08  (-1.26 to -0.90) | 0.007 | 0.021 | 0.814 |
| **BMI (kg/m^2^)** |  |  |  |  |  |  |  |  |  |  |  |  |  |
| Model 1 | -1.43  (-1.72 to -1.13) | -1.43  (-1.73 to -1.14) | 0.968 | 0.978 | -1.45  (-1.81 to -1.09) | -1.36  (-1.73 to -0.99) | 0.706 | 0.978 | -1.39  (-1.69 to -1.09) | -1.50  (-2.11 to -1.37) | 0.825 | 0.978 | 0.607 |
| Model 2 | -1.41  (-1.72 to -1.11) | -1.44  (-1.74 to -1.15) | 0.873 | 0.978 | -1.46  (-1.82 to -1.09) | -1.36  (-1.73 to -0.99) | 0.730 | 0.978 | -1.39  (-1.65 to -1.13) | -1.51  (-2.12 to -0.89) | 0.978 | 0.978 | 0.978 |

DASH, dietary approach to stop hypertension; FPG, fasting plasma glucose; WC, waist circumference; HOMA-IR, homeostatic model assessment for insulin resistance; Pi, P for interaction between TCF7L2 rs7903146 gene variant and intervention diets

Data for change in primary and secondary outcomes are express as mean (95% confidence interval)

Model 1 adjusted for baseline values

Model 2 adjusted for baseline values and oral anti-diabetic medications

P values were calculated by ANCOVA

Q value were calculated by Benjamini-Hochberg correction and Q<0.1 is significant
